# Protein Tyrosine Phosphatase 1B Inhibition and Glucose Uptake Potentials of Mulberrofuran G, Albanol B, and Kuwanon G from Root Bark of *Morus alba* L. in Insulin-Resistant HepG2 Cells: An In Vitro and In Silico Study

**DOI:** 10.3390/ijms19051542

**Published:** 2018-05-22

**Authors:** Pradeep Paudel, Ting Yu, Su Hui Seong, Eun Bi Kuk, Hyun Ah Jung, Jae Sue Choi

**Affiliations:** 1 Department of Food and Life Science, Pukyong National University, Busan 48513, Korea; phr.paudel@gmail.com (P.P.); yutdck@gmail.com (T.Y.); seongsuhui@naver.com (S.H.S.); 2 Department of Food Science and Human Nutrition, Chonbuk National University, Jeonju 54896, Korea; khkh55555@naver.com

**Keywords:** *Morus alba* L., root bark, protein tyrosine phosphatase 1B, α-Glucosidase, molecular docking, insulin-resistant HepG2

## Abstract

Type II diabetes mellitus (T2DM) is the most common form of diabetes and has become a major health problem across the world. The root bark of *Morus alba* L. is widely used in Traditional Chinese Medicine for treatment and management of diabetes. The aim of the present study was to evaluate the enzyme inhibitory potentials of three principle components, mulberrofuran G (**1**), albanol B (**2**), and kuwanon G (**3**) in *M. alba* root bark against diabetes, establish their enzyme kinetics, carry out a molecular docking simulation, and demonstrate the glucose uptake activity in insulin-resistant HepG2 cells. Compounds **1**–**3** showed potent mixed-type enzyme inhibition against protein tyrosine phosphatase 1B (PTP1B) and α-glucosidase. In particular, molecular docking simulations of **1**–**3** demonstrated negative binding energies in both enzymes. Moreover, **1**–**3** were non-toxic up to 5 µM concentration in HepG2 cells and enhanced glucose uptake significantly and decreased PTP1B expression in a dose-dependent manner in insulin-resistant HepG2 cells. Our overall results depict **1**–**3** from *M. alba* root bark as dual inhibitors of PTP1B and α-glucosidase enzymes, as well as insulin sensitizers. These active constituents in *M. alba* may potentially be utilized as an effective treatment for T2DM.

## 1. Introduction

Diabetes mellitus (DM) is a chronic disease that occurs when the pancreas is no longer able to produce insulin or when the body cannot take full advantage of its insulin. DM is a leading cause of cardiovascular disease, blindness, kidney failure, and lower limb amputation, since it affects the heart, blood vessels, eyes, kidneys, nerves, and teeth [1]. According to the 2015 International Diabetes Federation report, there are approximately 415 million adults with diabetes in the world, and this number will rise to 642 million by 2040. In 2015, five million deaths were attributed to DM, and it caused at least USD 673 billion in health expenditure. Therefore, DM has become a major health problem around the world. Type II DM (T2DM), characterized by resistance to insulin, is responsible for over 90% of the overall cases [2]. In the last several decades, numerous attempts have been made to find effective therapeutic drugs for DM, and the known therapeutic targets, α-glucosidase and protein tyrosine phosphatase 1B (PTP1B), have drawn the attention of many scientists. PTP1B plays a critical role in regulating glucose homeostasis and body weight by acting as a key negative regulator of insulin and the leptin signaling pathway, respectively [3]. In the intestinal lumen and brush border membrane, α-glucosidase plays a main role in carbohydrate digestion, and its inhibitors can prevent development of diabetes in people with impaired glucose tolerance and/or impaired fasting blood glucose [4]. Natural products, especially those used in Traditional Chinese Medicine (TCM), possess lower cytotoxicity and side effects than synthetic drugs and thus have become a subject of interest for scientists.

*Morus* (commonly known as mulberry) is a genus of flowering plants in the family Moraceae, which contains approximately 16 species. Used as fodder and traditional medicine, they are native to temperate regions and widely distributed in the subtropical regions of Asia, Africa, and the Americas [5,6]. The white mulberry (*M. alba* Linn) is cultivated to feed silkworms for commercial production of silk [7]. In TCM, *M. alba* root bark, twigs, leaves, and fruits have all been commonly used for centuries as liver tonics, to improve eyesight and lower blood pressure, and for treatment and management of disorders such as diabetes, arthritis, and fever [8,9]. The leaves are used as an anti-hyperglycemic supplement and are effective against high blood pressure. Phytochemicals such as terpenoids, alkaloids, flavonoids (including chalcones and anthocyanins), phenolic acids, stilbenoids, and coumarins have been identified in *M. alba* [10]. Many of these compounds exhibit various biological activities, including anti-oxidation, anti-inflammation, anti-fungal, anti-microbial, anti-tumor, anti-hypotension, and anti-diabetic activity [11,12,13]. Furthermore, studies on leaf extract from *M. alba* showed reduction in body weight, total cholesterol, triglycerides, and low-density lipoprotein level, as well as an antihyperlipidemic effect via the insulin receptor substrate 1 (IRS-1)/phosphoinositide 3-kinase (PI3K)/Glut-4 signaling pathway [14,15] and hypoglycemic potential through the increase of liver glucokinase activity and serum insulin level [16,17,18]. It was also reported that an *M. alba* leaf extract-containing diet could reduce insulin resistance and may delay the development of diabetes [19]. Fruit extract from *M. alba* improved hyperglycemia and insulin sensitivity via activation of the adenosine 5′-monophosphate (AMP)-activated protein kinase (AMPK) and AS160 in skeletal muscles and inhibition of gluconeogenesis in the liver [20]. Similarly, it was reported that a 70% alcohol extract of *M. alba* root bark could protect the generation of pancreatic β-cells, and a leaf extract could restore diminished β-cell numbers [21,22].

Interestingly, in a study conducted to evaluate the inhibitory activity of 266 kinds of herbs against α-glucosidase, root bark and leaves of *M. alba* displayed 96.5% and 93.6% inhibition, respectively [23]. Moreover, several polyhydroxylated alkaloids isolated from *M. alba* were found to be glucosidase inhibitors. Among them, a polyhydroxylated piperidine alkaloid, 1-deoxynojirimycin (DNJ), was the most potent [24]. In a recent report, morusin, licoflavone C, and mulberrofuran G isolated from the root bark of *M. alba* demonstrated significant α-glucosidase inhibition [25]. In addition, three flavonoids (kuwanon U, albanin D, and mortatarin D), monoterpenoid 2-arylbenzofurans, and geranylated 2-arylbenzofurans from the root bark of *M. alba* var. *tartarica* exhibited a significant degree of α-glucosidase inhibition, while albafuran A and albafuran B exhibited PTP1B inhibitory activity [26,27]. An investigation by Sarikaphuti et al. [28] revealed that anthocyanins extracted from *M. alba* are well tolerated and exhibit effective anti-diabetic properties in Zucker diabetic fatty rats. Anti-diabetic effects of various other constituents of *M. alba* leaves, such as DNJ, flavonoids and related compounds, polysaccharides, glycopeptides, and ecdysteroids have been reported. However, Hunyadi et al. [29] found that chlorogenic acid and rutin play a major role in anti-diabetic activity in vivo on type II diabetic rats, but knowledge about their contribution to the overall activity is limited. Thus, further research is necessary to discover other bioactive compounds responsible for anti-diabetic effects. Though the PTP1B and α-glucosidase inhibitory activity of mulberrofuran G (**1**) has recently been reported [25,30], there is no report for albanol B (**2**) and kuwanon G (**3**). Furthermore, this is the first attempt to demonstrate enzyme kinetics and simulate molecular docking of **1**–**3** against these two enzymes, and demonstrate glucose uptake activity in insulin-resistant HepG2 cells which is worthwhile to explore the pharmacological mechanism of *M. alba* in type II diabetes.

## 2. Results

### 2.1. Inhibitory Activity Against PTP1B and α-Glucosidase

**Figure 1:**
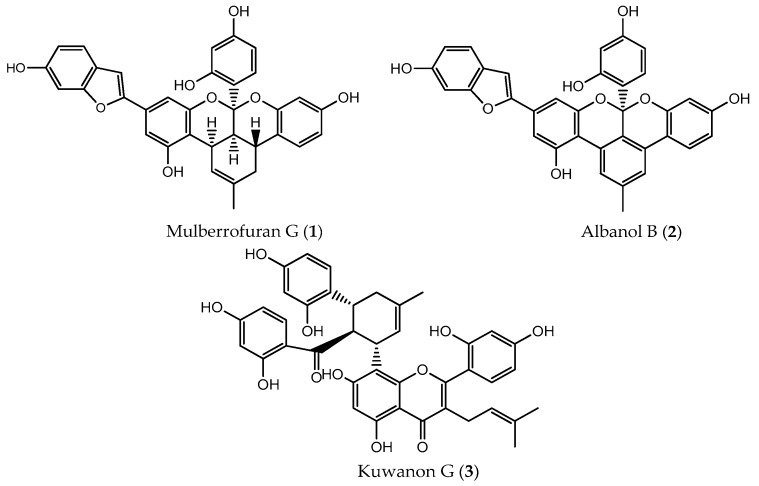
Structures of the isolated compounds from the root bark of *Morus alba* L.

The inhibitory potentials of **1**–**3** (Figure 1) against PTP1B and α-glucosidase were evaluated using *p*-nitrophenyl phosphate (*p*NPP) and *p*-nitrophenyl α-d-glucopyranoside (*p*NPG) as substrates, and the results are expressed as IC_50_ values (Table 1). All compounds showed higher PTP1B inhibitory activity than the positive control, ursolic acid (IC_50_, 3.54 ± 0.06 µM). Among them, **1** (IC_50_, 0.57 ± 0.04 µM) showed the highest inhibition, followed by **2** (IC_50_, 0.80 ± 0.02 µM) and **3** (IC_50_, 2.26 ± 0.03 µM).

Similarly, for α-glucosidase inhibition, all compounds were multifold more potent than the reference compound, acarbose (IC_50_, 119.16 ± 3.25 µM). In contrast to the PTP1B inhibition results, **2** (IC_50_, 1.31 ± 0.01 µM) showed the highest inhibition on α-glucosidase followed by **1** (IC_50_, 1.67 ± 0.02 µM) and **3** (IC_50_, 2.35 ± 0.03 µM).

### 2.2. Enzyme Kinetics of PTP1B and α-Glucosidase Inhibition

To explain the mode of enzymatic inhibition, kinetic analysis was performed at different concentrations of the substrates (*p*NPP for PTP1B and *p*NPG for α-glucosidase) and inhibitors. As shown in Table 1, **1**–**3** showed mixed type inhibition against both PTP1B (*K*_i_ values; 0.70, 1.02, and 1.98 μM, respectively) and α-glucosidase (*K*_i_ values; 1.20, 0.90, and 2.51 μM, respectively) (Figures S1 and S2). Since the *K*_i_ value represents the concentration needed to form an enzyme-inhibitor complex, a lower *K*_i_ value may indicate more effective inhibition against the enzyme system in the development of preventive and therapeutic agents.

### 2.3. Molecular Docking Simulation of PTP1B Inhibition

Investigation of protein-ligand interaction geometries at the molecular level can be accomplished via molecular docking simulations, a widely used computational approach. We performed molecular docking simulations of **1**–**3** with PTP1B using AutoDock 4.2 and validated the result using the reference ligands, 3-({5-[(*N*-acetyl-3-{4-[(carboxycarbonyl)(2-carboxyphenyl)amino]-1-naphthyl}-l-alanyl)amino]pentyl}oxy)-2-naphthoic acid (compound **23**) and (3-(3,5-dibromo-4-hydroxy-benzoyl)-2-ethyl-benzofuran-6-sulfonic acid 4-sulfamoyl-phenyl)-amide (compound **2**). A summary of the docking results regarding binding energies of test compounds and reference ligands, along with H-bond and hydrophobic interacting residues and the number of H-bonds, are presented in Table 2.

**Figure 2:**
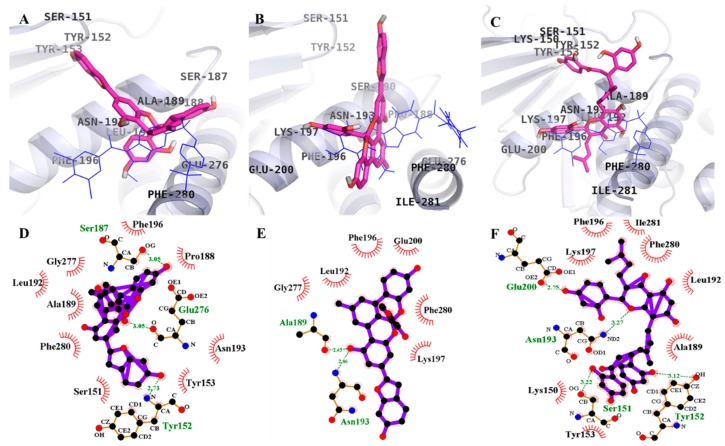
Molecular docking models for PTP1B inhibition at allosteric site by **1**–**3** along with reported inhibitor compound 2 (blue line). **1** (**A**); **2** (**B**); and **3** (**C**). 2D ligand interaction diagram of PTP1B inhibition by **1** (**D**); **2** (**E**); and **3** (**F**). Schematic representation of interaction between ligands **1**–**3** and the PTP1B, thick purple stick models present the compounds **1**–**3**, green dotted lines are hydrogen bonds, and dashed half-moons present hydrophobic interactions with the corresponding amino acid residues of the enzyme.

**Figure 3:**
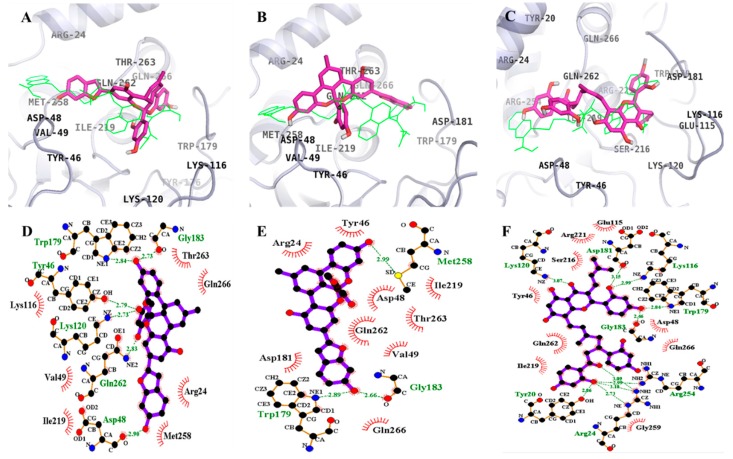
Molecular docking models for PTP1B inhibition at catalytic site by **1**–**3** along with reported inhibitor compound 23 (green line). **1** (**A**); **2** (**B**); and **3** (**C**). 2D ligand interaction diagram of PTP1B inhibition by **1** (**D**); **2** (**E**); and **3** (**F**). Schematic representation of interaction between ligands **1**–**3** and the PTP1B, thick purple stick models present the compounds **1**–**3**, green dotted lines are hydrogen bonds, and dashed half-moons present hydrophobic interactions with the corresponding amino acid residues of the enzyme.

Simulation results are shown in Figure 2 and Figure 3. The PTP1B-**1** inhibitor complex at the allosteric site showed a −8.24 kcal/mol binding energy with three hydrogen bonds and interacting residues of Ser187, Glu276, and Tyr152. As illustrated in Figure 2D, bond distances between **1** and the interacting amino acid residues were 3.05 Å for Ser187 and Glu276 and 2.73 Å for Tyr152. In addition, hydrophobic interactions were also observed between Phe196, Pro188, Asn193, Tyr153, Ser151, Phe280, Ala189, Leu192, and Gly277.

Catalytic inhibition by **1** against PTP1B (−6.85 kcal/mol) displayed six H-bonds with the residues Trp179, Tyr46, Lys120, Gln262, Asp48, and Gly183 and hydrophobic interactions between Thr263, Gln266, Arg24, Met258, Ile219, Val49, and Lys116 (Figure 3D). Moreover, the binding energies of respective reference ligands, compound **2** and compound **23**, at their respective binding sites were −10.98 and −11.23 kcal/mol. The PTP1B-**2** inhibitor complex at the allosteric site showed a −7.37 kcal/mol binding energy with two hydrogen bonds and interacting residues of Asn193 and Ala189. As illustrated in Figure 2E, bond distances between **2** and the respective interacting amino acid residues were 2.86 and 2.65 Å, respectively. In addition, hydrophobic interactions were also observed between Gly277, Leu192, Phe196, Glu200, Phe280, and Lys197. Displaying three H-bonds with the residues Met258, Gly183, and Trp179 and hydrophobic interactions between Arg24, Tyr46, Ile219, Asp48, Thr263, Gln262, Val149, Gln266, and Asp181 (Figure 3E), **2** exhibited catalytic inhibition against PTP1B (−6.70 kcal/mol). The PTP1B-**3** inhibitor complex at the allosteric site showed a −7.81 kcal/mol binding energy with four hydrogen bonds and interacting residues of Glu200, Asn193, Ser151, and Tyr152. As illustrated in Figure 2F, bond distances between **3** and the respective interacting amino acid residues were 2.75, 3.27, 3.22, and 3.12 Å along with hydrophobic interactions between Lys197, Phe196, Ile281, Phe280, Leu192, Ala189, Tyr153, and Lys150. However, at the catalytic site (−7.62 kcal/mol), **3** demonstrated eight H-bonds with the residues Asp181, Lys120, Lys116, Trp179, Gly183, Arg254, Tyr20, and Arg24 and hydrophobic interactions between Glu115, Arg221, Ser216, Tyr46, Gln262, Asp48, Gln266, Gly259, and Ile219 (Figure 3F). Overall, the results of the docking study concur with those of the enzyme kinetics study unveiling mixed type inhibition.

### 2.4. Molecular Docking Simulation of α-Glucosidase Inhibition

**Figure 4:**
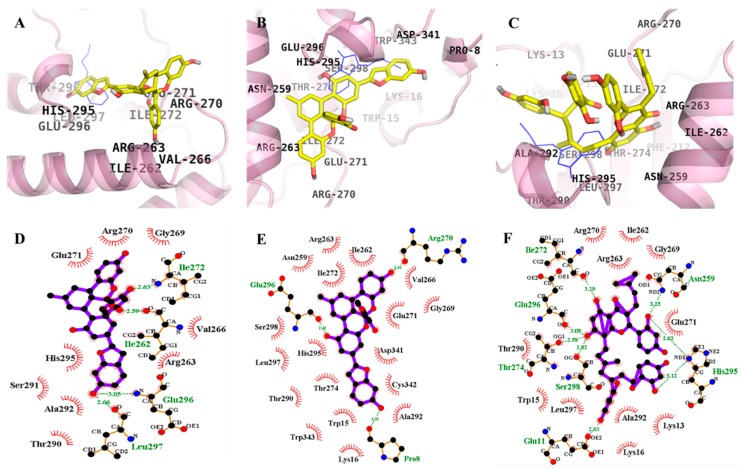
Molecular docking models for α-glucosidase inhibition at allosteric site by compounds **1**–**3** along with reported inhibitor BIP (blue line). **1** (**A**); **2** (**B**); and **3** (**C**). 2D ligand interaction diagram of α-glucosidase inhibition by **1** (**D**); **2** (**E**); and **3** (**F**). Schematic representation of interaction between ligands **1**–**3** and the α-glucosidase, thick purple stick models present the compounds **1**–**3**, green dotted lines are hydrogen bonds, and dashed half-moons present hydrophobic interactions with the corresponding amino acid residues of the enzyme.

**Figure 5:**
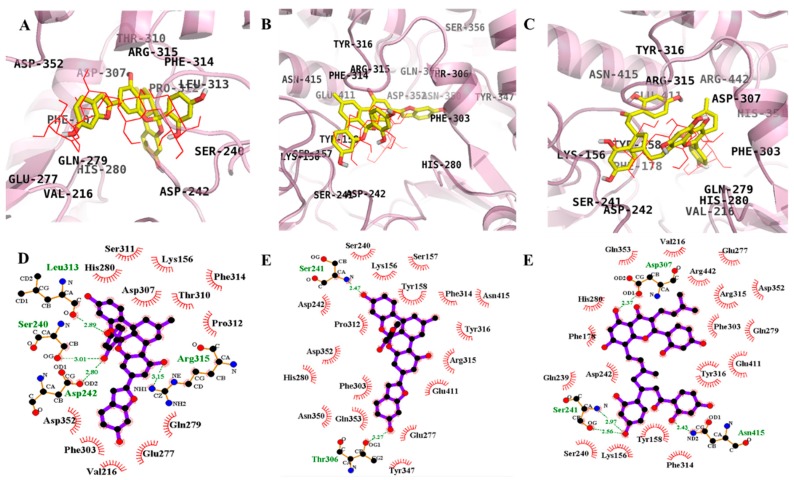
Molecular docking models for α-glucosidase inhibition at catalytic site by compounds **1**–**3** along with reported inhibitor acarbose (red line). **1** (**A**); **2** (**B**); and **3** (**C**). 2D ligand interaction diagram of α-glucosidase inhibition by **1** (**D**); **2** (**E**); and **3** (**F**). Schematic representation of interaction between ligands **1**–**3** and the α-glucosidase, thick purple stick models present the compounds **1**–**3**, green dotted lines are hydrogen bonds, and dashed half-moons present hydrophobic interactions with the corresponding amino acid residues of the enzyme.

Molecular docking simulations of **1**–**3** with α-glucosidase were performed, and the ligand–enzyme complexes of the three test compounds/or acarbose and (*Z*)-3-butylidenephthalide (BIP) were stably posed in the same pocket of the α-glucosidases by AutoDock 4.2 (Figure 4 and Figure 5). Binding energies of the test compounds with interacting residues, including H-bond interacting residues and hydrophobic interacting residues, along with the number of H-bonds are listed in Table 3. With −8.65 kcal/mol binding energy, **1** showed allosteric inhibition forming four hydrogen bonds with interacting residues Ile262, Ile272, Glu296, and Leu297. As illustrated in Figure 4D, bond distances between **1** and the respective interacting amino acid residues were 2.50, 2.03, 3.05, and 2.66 Å. In addition, hydrophobic interactions were also observed between Arg263, Val266, Gly269, Arg270, Glu271, Thr290, Ser291, Ala292, and His295.

However, **1** exhibited catalytic inhibition against α-glucosidase with least binding energy (−10.43 kcal/mol), displaying four H-bonds with the residues Ser240, Asp242, Leu313, and Arg315 and hydrophobic interactions between His280, Ser311, Lys156, Asp307, Thr310, Pro312, Gln279, Glu277, Val216, Phe303, Asp352, and Phe314 (Figure 5D). Similarly, the α-glucosidase-**2** inhibitor complex at the allosteric site showed a −11.71 kcal/mol binding energy with three hydrogen bonds and interacting residues of Pro8, Arg270, and Glu296. As illustrated in Figure 4E, bond distances between **2** and the respective interacting amino acid residues were 3.02, 2.42, and 2.42 Å. In addition, hydrophobic interactions were also observed for Trp15, Lys16, Thr274, Thr290, His295, Leu297, Ser298, Trp343, Cys342, Ala292, Asp341, Glu271, Gly269, Val266, Ile262, Ile272, Asn259, and Arg263.

While for the catalytic inhibition (−9.48 kcal/mol), **2** displayed two H-bonds with the residues Ser241 and Thr306 and hydrophobic interactions between Lys156, Ser157, Tyr158, Glu227, Ser240, Asp242, His280, Phe303, Pro312, Phe314, Arg315, Tyr316, Tyr347 Asn350, Asp352, Gln353, Glu411, and Asn415 (Figure 5E). With eight hydrogen bonds and interacting residues Ile272, Glu296, Thr274, Glu11, His295, Asn259, and Ser298, **3** showed allosteric inhibition with −7.36 kcal/mol binding energy. As illustrated in Figure 4F, bond distances between **3** and the respective interacting amino acid residues were 3.29, 3.09, 2.58, 2.03, 2.63, 3.25, and 2.82 Å. In addition, hydrophobic interactions were observed between Arg270, Ile262, Arg263, Gly269, Glu271, Lys13, Ala292, Lys16, Leu297, Trp15, and The290. However, with a binding energy −11.53 kcal/mol and four H-bonds with the residues Ser241, Asp307, and Asn415, **3** displayed catalytic inhibition along with hydrophobic interactions between Lys156, Tyr158, Phe178, Val216, Gln239, Ser240, Asp242, Glu277, Gln279, His280, Phe303, Phe314, Arg315, Tyr316, Asp351, Gln353, Glu411, and Arg442 (Figure 5F).

As shown in Table 3, the docking results of a known α-glucosidase catalytic inhibitor, acarbose, formed 17 hydrogen bonds with interacting Tyr158, His112, Gln182, Asp69, Asp215, Arg213, Glu277, Asp352, Arg442, Asp307, His280, Asp242, and Ser240 residues, while BIP, a potent allosteric inhibitor of α-glucosidase, formed two hydrogen bonds with interacting Glu296 and His295 residues.

### 2.5. Evaluation of Cytotoxicity in HepG2 Cells

**Figure 6:**
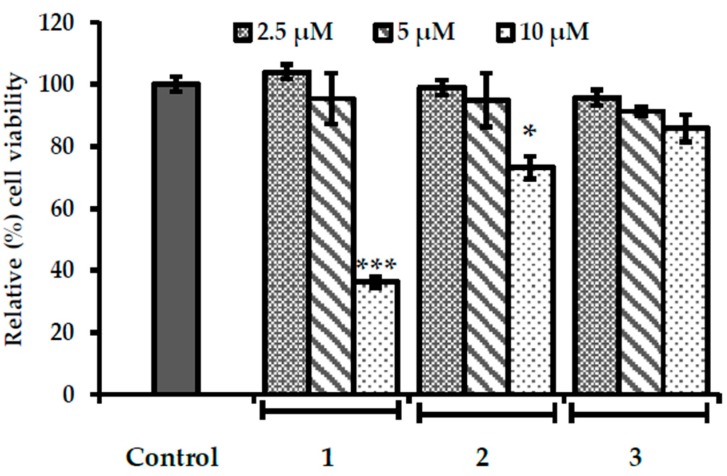
Effects of **1**–**3** on cell viability in HepG2 cells measured by MTT assay. Data shown represent means ± standard deviation of triplicate experiments. * *p* < 0.01 and *** *p* < 0.001 indicate significant differences from the control group.

In order to determine safe concentrations for cell experiments, the cytotoxicity of our test compounds in HepG2 cells was evaluated using an 3-(4,5-dimethylthiazol-2-yl)-2,5-diphenyltetrazolium bromide (MTT) assay. As shown in Figure 6, all compounds were non-toxic up to 5 µM (viable cell %; 90–105). However, at 10 µM, the level of toxicity was high for **1** (viable cell %; 36), followed by **2** (viable cell %; 73) and **3** (viable cell %; 85). Therefore, 1.25, 2.5, and 5 µM concentrations were used in further cell experiments.

### 2.6. Effects on Glucose Uptake in Insulin-Resistant HepG2 Cells

**Figure 7:**
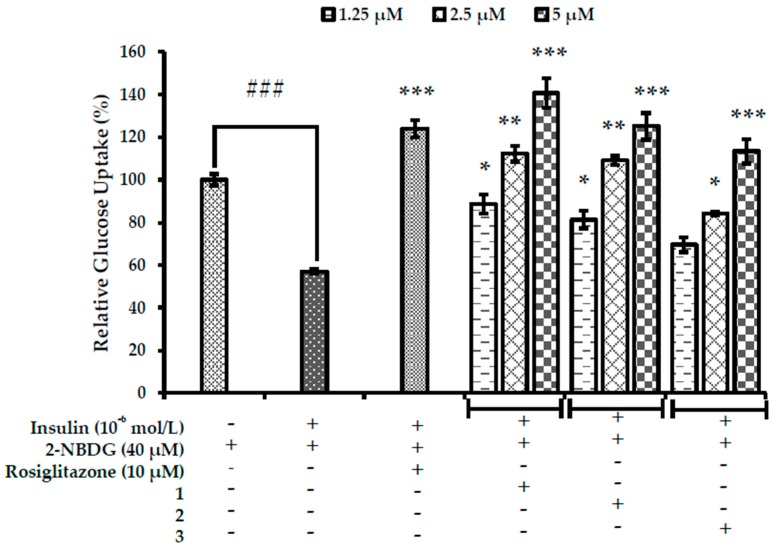
Effects of **1**–**3** on insulin stimulated glucose uptake in insulin-resistant HepG2 cells as measured by 2-[*N*-(7-nitrobenz-2-oxa-1,3-diazol-4-yl) amino]-2-deoxyglucose (2-NBDG) method. Data shown represent means ± standard deviation of triplicate experiments. ^###^ *p* < 0.001 indicates significant differences from the control group; * *p* < 0.05, ** *p* < 0.01 and *** *p* < 0.001 indicate significant differences from the 10^−6^ M insulin-treated control group.

A 2-[*N*-(7-nitrobenz-2-oxa-1,3-diazol-4-yl) amino]-2-deoxyglucose (2-NBDG) test was used to measure glucose uptake in insulin-resistant HepG2 cells. Treatment of HepG2 cells with high dose insulin induced insulin resistance as indicated by the marked reduction in glucose uptake in insulin-resistant group (Figure 7). However, all test compounds significantly increased insulin-stimulated 2-NBDG uptake in insulin-resistant HepG2 cells in a dose-dependent manner. Among them, **1** showed the most potent effect, followed by **2** and **3**.

The relative glucose uptake percentages of **1** and **2** at 5 µM concentrations were 140% and 125%, which were higher than the positive control, rosiglitazone (relative % glucose uptake; 123% at 10 µM). Considering the relative % glucose uptake activity of rosiglitazone at 10 µM and test compounds at 5 µM in insulin-resistant HepG2 cells, our test compounds are more potent. All these results suggests that **1**–**3** from root bark of *M. alba* are able to induce sensitivity of insulin-resistant HepG2 cells towards insulin thereby increasing the relative percentage glucose uptake.

### 2.7. Effects on PTP1B Expression Level in Insulin-Resistant HepG2 Cells

**Figure 8:**
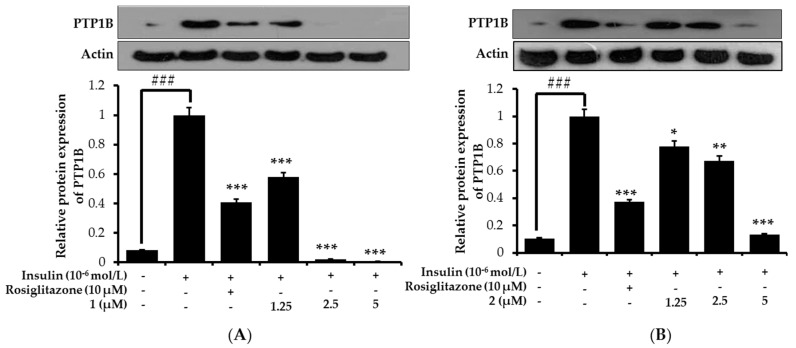

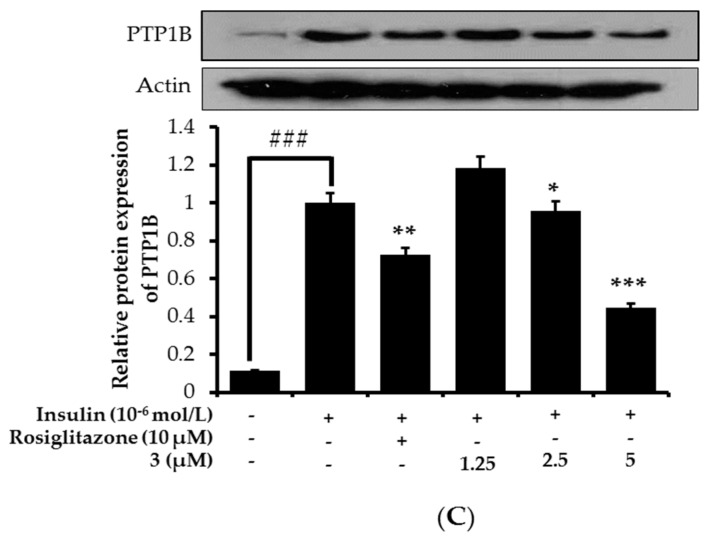
Effects of **1** (**A**); **2** (**B**); and **3** (**C**) on protein tyrosine phosphatase 1B (PTP1B) expression level in insulin-resistant HepG2 cells. Western blotting was performed and protein band intensities were quantified by densitometric analysis. Upper panels display representative blots. Equal protein loading was ensured and normalized against β-actin levels. Values are the mean ± standard deviation of three independent experiments; ^###^ *p* < 0.001 indicates significant differences from the control group; * *p* < 0.05, ** *p* < 0.01 and *** *p* < 0.001 indicate significant differences from the 10^−6^ M insulin-treated control group.

PTP1B is a negative regulator of insulin signaling, and its high expression is implicated in insulin resistance. Therefore, in order to confirm whether these compounds from *M. alba* increase insulin sensitivity by inhibiting PTP1B expression, we evaluated the expression in insulin-resistant HepG2 cells by Western blot. Under normal condition, PTP1B is expressed feebly while it is intensified in insulin-resistant state. So the downregulation of PTP1B expression in insulin-resistant HepG2 cells could be the indication of insulin sensitizing property. As shown in Figure 8, all three compounds inhibited PTP1B expression in a dose-dependent manner, disclosing their insulin-sensitizing potential, which was in accordance with the glucose uptake activity of respective compounds.

At 2.5 and 5 μM concentration, **1** diminished the PTP1B expression almost completely. While **2** showed the expression that was comparable to the normal group at 5 μM concentration. Although **3** could not normalize the condition completely, it downregulated the expression towards normal in a concentration dependent manner. Overall result of PTP1B downregulation by **1**–**3** in insulin-resistant HepG2 cells coincide with their PTP1B enzyme inhibition results.

## 3. Discussion

T2DM is the most common form of DM. It occurs in quick succession and is characterized by inadequacy in insulin secretion and resistance to insulin in its target organs. Several hypoglycemic agents with different mechanisms of action, such as increasing production of insulin, decreasing production of hepatic glucose, limiting absorption of postprandial glucose, and inhibiting gluconeogenesis, have been developed. However, periodic diminishment in efficacy of single and even combined drugs leads to the administration of insulin [31].

The association of insulin resistance with obesity and T2DM and the fact that insulin receptor signaling is mediated by tyrosine (Tyr) phosphorylation have generated great interest in the homeostasis of tyrosine phosphorylation. Among several implicated Tyr phosphatases, PTP1B is the most prominent. Therefore, targeting PTP1B and developing novel PTP1B inhibitors could lead to an effective therapeutic model against diabetes via insulin sensitization, without the weight gain associated with thiazolidinediones [32]. However, use of available synthetic PTP1B inhibitors has been limited due to their dose-dependent side effects and failure in Phase II clinical trials. In addition, natural molecules with improved pharmacodynamics/pharmacokinetic parameters can have advantages over synthetic molecules. The other approach to treating diabetes, particularly non-insulin-dependent diabetes (postprandial hyperglycemia), is to retard glucose absorption via inhibition of the carbohydrate hydrolyzing enzyme α-glucosidase. α-Glucosidase is the principle enzyme in the catalyzation of the final step of carbohydrate metabolism. Inhibiting this enzyme retards the formation of d-glucose from dietary carbohydrates, delays glucose absorption/reduces postprandial plasma glucose level, and finally suppresses postprandial hyperglycemia. The existing clinical α-glucosidase inhibitors (acarbose, miglitol, and voglibose) are associated with gastrointestinal side effects [33]. Therefore, discovery of novel phytochemicals with potent PTP1B and α-glucosidase inhibition with no or fewer side effects is urgent.

*Morus alba* L. is a well-known TCM herb with a decades-long history of medicinal value. Various parts of this plant (fruits, leaves, branches, and root bark) have been used for medicinal purposes. As part of our ongoing work to discover novel phytochemicals against diabetes, we evaluated the inhibitory potentials of **1**–**3** that we isolated from root bark of this plant [34] against PTP1B and α-glucosidase. All compounds showed potent inhibition against both enzymes and were superior to reference drugs. Further, in order to validate our enzyme inhibition result, we performed enzyme kinetics. Enzyme kinetic analyses performed with various concentrations of substrates and inhibitors showed mixed-type inhibition against PTP1B and α-glucosidase, implying that these compounds bind to the active site as well as an additional binding site of the respective enzymes. The findings of this study demonstrate that **1**–**3** might act as preventive or therapeutic agents for type II diabetes by inhibiting PTP1B and α-glucosidase enzyme activity. Because lower *K*_i_ values indicate tighter binding to the enzyme, the low *K*_i_ values of these compounds signify that they could be highly effective against PTP1B and α-glucosidase.

Structure-based drug design is one of various vital computational approaches found to be effective in identification of hits for in vitro testing. Molecular docking is an extensively used method of virtually screening small molecules for identification and optimization. Additionally, by using docking score functions, one can accurately predict the binding affinities between ligands and receptors and understand the mechanism behind binding sites interactions. Structural and kinetic analyses have identified two important amino acids, Cys215 in the PTP signature motif (P-loop) and Asp181 in the WPD loop, as the primary active site residues that attack substrates. Once the active sites are blocked by the WPD loop upon inhibitor/substrate binding, dephosphorylation of the phosphocenter of the substrate by Cys215 occurs via nucleophilic attack. Similarly, Arg47, Lys120, and Val49 demonstrate electrostatic, hydrophobic, and H-bonding interactions [35]. In addition, Puius et al. reported the existence of second catalytic site (“site B”) in which Arg24 and Arg254 were the important residues including H-bond interactions with Met258 and Gln262, and van der Waals contact with Ile219, Asp48, and Val49 [36]. So, to confirm the inhibition mode of enzyme inhibition, we further conducted molecular docking studies and validated our results using reference ligands (compound **2** as allosteric and compound **23** as catalytic inhibitors).

Molecular docking studies on PTP1B inhibition revealed that inhibition by the reference allosteric inhibitor, compound 2, occurred via Asn193 and Glu276. **1** showed interaction with Glu276 while, **2** and **3** interacted with Asn193 for allosteric inhibition. In addition, for catalytic inhibition, the mode of interaction was through Arg24, Arg254, Asp48, and Tyr46 along with additional amino acid residues compared with the reference inhibitor, compound **23** (Table 2). Since low binding energy indicates high binding affinity, **1**–**3** showed preference for allosteric inhibition. The enzyme kinetic study revealed **1**–**3** as mixed inhibitors; however, the molecular docking study was more specific and concluded the inhibition to be mixed but tending toward allosteric mode. Similarly, for α-glucosidase, **1**–**3** showed low binding energies for catalytic inhibition compared to allosteric inhibition (Table 3), which indicates that these compounds bind preferentially to catalytic sites. This means the test compound mode of inhibition for α-glucosidase was mixed but tending toward catalytic inhibition.

To further investigate the anti-diabetic mechanisms of the test compounds, we evaluated the effect of these compounds on insulin sensitivity in normal versus insulin-resistant HepG2 cells. The insulin-resistant HepG2 cell model is widely used in the field of drug discovery for evaluating anti-insulin resistance bioactivity from plant resources. PTP1B is a well-known negative regulator of the insulin signaling pathway, and its expression is elevated in insulin-resistant conditions [37]. The biological effect of insulin is initiated with insulin binding to the α-subunit of IR and activating the intrinsic tyrosine kinase activity of the β-subunit of the receptor. The activation of IR leads to tyrosine phosphorylation of IRS. Thus phosphorylated IRS then activates PI3K, which subsequently phosphorylates Ser/Thr kinase Akt. Beside this, activation of Akt promotes the translocation of intracellular GLUT4 to plasma membrane and lead to glucose transport. However, in case of insulin resistance state, cell become unresponsive to insulin as a result of which insulin signaling cascade get dysregulated. Studies have shown that overexpression of PTP1B inhibits IR and IRS phosphorylation and leads to insulin resistance [38,39]. In a recent report, PTP1B inhibitor increased the tyrosine phosphorylation of IR, and activated the downstream molecules of insulin signaling such as IRS-1, Akt, and Erk1/2 [40,41]. Similarly, a study on fumosorinone as a novel PTP1B inhibitor showed decreased PTP1B expression, that led to increased phosphorylation of IRβ, IRS-2, Akt, GSK3β and Erk1/2 in insulin-resistant HepG2 cells along with the increased phosphorylation of IRβ, IRS-2 and Akt in diabetic KKAy mice [42]. Increased glucose uptake in insulin-resistant HepG2 cells treated with sample compared to the untreated insulin-resistant/normal group confirms improvement of insulin sensitivity. In the 2-NBDG assay of present study, **1**–**3** increased insulin-stimulated glucose uptake significantly in a dose-dependent manner. Similarly, in Western blot analysis, all the test compounds suppressed PTP1B expression in a concentration-dependent manner. PTP1B was feebly expressed in the normal group but intensified in the insulin-resistant group. However, its expression declined in the rosiglitazone- and sample-treated groups. Though PTP1B inhibition is recognized as an effective way to improve insulin sensitivity, better understanding of the PTP1B-regulatory mechanism is still lacking. Dysregulation of miRNA-122 contributes to hepatic insulin resistance through PTP1B induction. So, recovering the expression of miRNA-122 down regulate PTP1B and abolish hepatic insulin resistance [43]. In in vitro and in cells, PTP1B recognizes the activated insulin receptor as a substrate and preferentially dephosphorylates tyrosine residue in the activation loop of the β-subunit of insulin receptor. Additionally, IRS-1 is also a potential substrate of PTP1B. The pathology of hyperinsulinemia-induced insulin resistance is associated with impaired Ser/Tyr phosphorylation of IRS-1 and IRS-2 that impairs their interactions with cytoplasmic domain of insulin receptors and deregulate the insulin signaling. As a result, in insulin-resistant condition, PTP1B is highly expressed. However, treatment with PTP1B inhibitors regulate the Ser/Tyr phosphorylation of IRS-1 and IRS-2 thereby down regulating PTP1B expression. Therefore, improvement of insulin sensitivity of high insulin-induced insulin-resistant HepG2 by **1**–**3** might be via regulation of Ser/Tyr phosphorylation of IRS-1 and IRS-2 in insulin-signaling cascade. Besides, activation of Akt and translocation of intracellular GLUT4 to plasma membrane might also be the underlying mechanism of enhanced glucose uptake by these compounds.

Our results in overall reveal that **1**–**3** from *M. alba* and rosiglitazone improve insulin sensitivity by suppressing PTP1B expression. In conclusion, *Morus alba* root bark contains a familiar TCM herb that has been widely used for various biological disorders, especially T2DM. Notably, our study provided systematic and scientific evidence supporting its pharmacological and therapeutic effects in diabetes. Overall results showed the usefulness of **1**–**3** from *M. alba* root bark in the management of T2DM through an increase in the sensitivity of insulin-resistant cells to insulin and an enhancement in glucose uptake via PTP1B down-regulation. However, possible involvement of **1**–**3** in other mechanisms for improvement of diabetic conditions should be studied further.

## 4. Materials and Methods

### 4.1. Chemicals and Reagents

Mulberrofuran G, albanol B, and kuwanon G were isolated from the EtOAc fraction of *M. alba* root bark as previously described [34] and identified by direct comparison with authentic samples (^1^H and ^13^C-NMR). The structures of **1**–**3** are shown in Figure 1. Ethylenediaminetetraacetic acid (EDTA), *p*-nitrophenyl phosphate (*p*NPP), rosiglitazone, and DMSO were purchased from Sigma-Aldrich Co. (St. Louis, MO, USA). PTP1B (human recombinant) was purchased from Biomol International LP (Plymouth Meeting, PA, USA). Fetal bovine serum (FBS), minimum essential medium (MEM), sodium pyruvate, penicillin-streptomycin, and nonessential amino acids were purchased from Gibco-BRL Life Technologies (Grand Island, NY, USA). The fluorescent d-glucose analogue and glucose tracer 2-[*N*-(7-nitrobenz-2-oxa-1,3-diazol-4-yl) amino]-2-deoxy-d-glucose (2-NBDG) was purchased from Life Technologies (Carlsbad, CA, USA). Human insulin was purchased from Eli Lilly (Fegersheim, France). All other chemicals and solvents used were of reagent grade and acquired from commercial sources.

### 4.2. Protein Tyrosine Phosphate 1B (PTP1B) Inhibitory Assay

PTP1B inhibitory activity was evaluated using *p*NPP [44]. Recombinant PTP1B enzyme (0.5 units diluted in PTP1B reaction buffer) was added to a plate with or without sample. The plate was pre-incubated at 37 °C for 10 min, and then substrate (2 mM *p*NPP) was added. Following incubation at 37 °C for 15 min, the enzymatic reaction was terminated by addition of 10 M NaOH. The absorbance was measured at 405 nm using a microplate spectrophotometer (Molecular Devices, Sunnyvale, CA, USA). Ursolic acid was used as a reference compound.

### 4.3. α-Glucosidase Inhibitory Assay

The enzyme inhibition study was carried out spectrophotometrically using the procedure previously reported [45]. The α-glucosidase activity was determined by measuring release of *p*NPG at 405 nm using a microplate spectrophotometer (Molecular Devices). Acarbose was used as a reference compound.

### 4.4. Kinetic Study Against PTP1B and α-Glucosidase

To determine the modes of enzyme inhibition, enzymatic inhibition of the test samples was evaluated by monitoring the effects of different concentrations of the substrates (0.5, 1, or 2 mM *p*NPP for PTP1B and 0.4, 0.8 or 1.6 mM *p*NPG for α-glucosidase) in the Dixon plots (single reciprocal plots). Lineweaver–Burk plots for inhibition of PTP1B were obtained in the presence of various concentrations of the test compounds (0, 0.18, 0.44 and 1.78 μM for **1**; 0, 0.27, 0.54, and 1.07 μM for **2**; 0, 0.87, 1.44, and 2.89 μM for **3**). Similarly, for the inhibition of α-glucosidase, text concentrations used were 0, 0.71, 1.07, and 1.78 μM for **1**; 0, 0.36, 0.9, and 1.79 μM for **2** and 0, 0.72, 2.88, and 5.77 μM for **3**. The enzymatic procedure consisted of the same aforementioned assay method. The inhibition constants (*K*_i_) were determined via interpretation of the Dixon plots, where the value of the *x*-axis implies −*K*_i_ [46,47].

### 4.5. Molecular Docking Simulation of PTP1B and α-Glucosidase Inhibition

The structure of PTP1B complexed with its selective allosteric inhibitor 3-(3,5-dibromo-4-hydroxy-benzoyl)-2-ethyl-benzofuran-6-sulfonic acid (4-sulfamoyl-phenyl)-amide (compound **2**) (PDB ID: 1T49) and the 3D structure of catalytic inhibitor 3-({5-[(*N*-acetyl-3-{4-[(carboxycarbonyl)(2-carboxyphenyl)amino]-1-naphthyl}-l-alanyl)amino]pentyl}oxy)-2-naphthoic acid (compound **23**) were obtained from the RCSB Protein Data Bank website [48] and PubChem Compound (NCBI, Bethesda, MD, USA) with a compound CID of 447410. The structure of α-glucosidase with its catalytic ligand α-d-glucose (PDB ID: 3A4A) and the structures of acarbose and (*Z*)-3-butylidenephthalide (BIP) were obtained from the RCSB Protein Data Bank website [49] and PubChem Compound (NCBI) with compound CIDs of 41774 and 5352899, respectively. Protein preparation was conducted using Accelrys Discovery Studio 16.1 (Accelrys, Inc., San Diego, CA, USA). The binding areas of compound **23**, compound **2**, acarbose, and BIP were considered to be the most convenient regions for ligand binding in the docking simulation. The 3D structures of mulberrofuran G (PubChem CID: 9959532), albanol B (PubChem CID: 480819), and kuwanon G (PubChem CID: 5281667) were obtained from PubChem Compound (NCBI) and protonated using MarvinSketch (ChemAxon, Budapest, Hungary). A Lamarckian genetic algorithm (GA) method was used for docking. Gasteiger charges were added by default, the rotatable bonds were set with ADT, and all torsions were allowed to rotate. Grid box size was set to maximum with default spacing. The docking simulation was conducted with 10 independent GAs with the default parameters. Results were analyzed using UCSF Chimera (Available online: http://www.cgl.ucsf.edu/chimera/), while the hydrogen bond interacting residues and hydrophobic interacting residues were visualized using LigPlot^+^.

### 4.6. Cell Culture, MTT Assay and Insulin Resistance Induction

HepG2 (human hepatocarcinoma) cells, purchased from the American Type Culture Collection (HB-8065; Manassas, VA, USA), were maintained in 10% FBS MEM at 37 °C in a humidified atmosphere with 5% CO_2_. Medium was changed every 48 h. Cytotoxicity was evaluated using the MTT assay [50]. Following the method previously described [42], insulin-resistant HepG2 cell model was established. All the other experiment conditions and procedures were similar to those reported in our previous paper [51].

### 4.7. Glucose Uptake Assay

The glucose uptake rate in insulin-resistant HepG2 cells was assessed using the fluorescent d-glucose analog 2-NBDG. Once the induction of insulin resistance was accomplished, cell solutions in the wells were substituted with different concentrations of **1**–**3** or a positive control, rosiglitazone (10 µM), prepared in serum-free MEM (SFMEM) in respective groups. After 24 h incubation with samples, the media in the wells was replaced with 100 nM insulin diluted in SFMEM and incubated for 30 min. Cells were then incubated with 40 μM 2-NBDG for 30 min and washed with ice-cold PBS. The fluorescence intensity of 2-NBDG was measured on a fluorescence microplate reader (FL ×800, Bio-Tek Instruments, Inc., Winooski, VT, USA) at 485 nm excitation and 528 nm emission wavelengths. Six replicate wells were established, and each experiment was repeated three times.

### 4.8. Preparation of Cell Lysates and Western Blot Analysis

Insulin-resistant HepG2 cells were exposed to different concentrations of **1**–**3** or rosiglitazone (10 μM) in 24-well plates. After 24 h of incubation with samples, the media in the wells was replaced with 100 nM insulin diluted in SFMEM and incubated at 37 °C for 30 min. Cells were then washed twice with ice-cold PBS, collected, and lysed with sample buffer (50 mM HEPES, pH 7.5, 150 mM NaCl, 2.5 mM EDTA, 0.5% NP-40, 1 mM PMSF, 1 mM DTT, 0.2% aprotinin, 0.5% leupeptin, 20 mM NaF, and 1 mM Na_3_VO_4_). Protein was quantified by the modified Bradford protein assay kit using BSA as a standard. Total protein (50 μg) was electrophoresed on sodium dodecyl sulfate-polyacrylamide gels (Bio-Rad, Hercules, CA, USA) and then transferred to polyvinylidene difluoride (PVDF) membranes (Immobilon-P; Millipore, Burlington, MA, USA) using the Pierce G2 Fast Blotter (Thermo Fisher Scientific Inc., Fair Lawn, NJ, USA) at 100 V for 60 min in a standard semi-dry system. Membranes were blocked in blocking buffer (5% skim milk powder in TBS-Tween buffer containing 10 mM Tris, 100 mM NaCl, and 0.1% Tween-20, pH 7.4) and incubated overnight with primary antibodies at 4 °C on a shaker, followed by incubation with appropriate secondary antibodies for 2 h at room temperature. The membranes were washed three times with TBST (3 × 10 min each) and the protein bands were visualized with the Supersignal West Pico Chemiluminescence Substrate (Pierce, Rockford, IL, USA) on X-ray films (Kodak, Rochester, NY, USA) and quantitated using CS analyzer software (Atto Corp., Tokyo, Japan).

### 4.9. Statistical Analysis

One-way analysis of variance (ANOVA) and Student’s *t*-test (Systat Inc., Evanston, IL, USA) were used to determine the statistical significance. A *p*-value < 0.01 was considered significant. All the results are presented as the mean ± standard error of the mean (SEM).

## Figures and Tables

**Table 1:** Protein tyrosine phosphatase 1B and α-glucosidase inhibitory activity of the compounds isolated from *M. alba*.

Compounds	Protein Tyrosine Phosphatase 1B			α-Glucosidase		
	IC_50_ (μM) ^a^	Inhibition Type ^b^	*K*_i_ (μM) ^c^	IC_50_ (μM) ^a^	Inhibition Type ^b^	*K*_i_ (μM) ^c^
**1**	0.57 ± 0.04	Mixed type	0.70	1.67 ± 0.02	Mixed type	1.2
**2**	0.80 ± 0.02	Mixed type	1.02	1.31 ± 0.01	Mixed type	0.9
**3**	2.26 ± 0.03	Mixed type	1.98	2.35 ± 0.03	Mixed type	2.51
Ursolic acid ^d^	3.54 ± 0.06					
Acarbose ^e^				119.16 ± 3.25		

^a^ The 50% inhibitory concentration (µM) was calculated from a log-dose inhibition curve and is expressed as the mean ± standard error of the mean (SEM) of triplicate experiments; ^b^ Inhibition type was determined by interpretation of the Lineweaver-Burk plot; ^c^ The inhibition constant (K_i_) was determined by interpretation of the Dixon plot; ^d, e^ Positive controls used in respective assays.

**Table 2:** Molecular interaction of the protein tyrosine phosphatase 1B (PTP1B) active site with **1**–**3** as well as reference inhibitors.

Compound	Binding Energy ^a^ (kcal/mol)	No. of H-Bond ^b^	H-Bond Interacting Residues ^c^	Hydrophobic Interacting Residues ^d^
**1** (Allosteric inhibition mode)	–8.24	3	Ser187, Glu276, Tyr152	Phe196, Pro188, Asn193, Tyr153, Ser151, Phe280, Ala189, Leu192, Gly277
**1** (Catalytic inhibition mode)	–6.85	6	Trp179, Tyr46, Lys120, Gln262, Asp48, Gly183	Thr263, Gln266, Arg24, Met258, Ile219, Val49, Lys116
**2** (Allosteric inhibition mode)	–7.37	2	Ala189, Asn193	Gly277, Leu192, Phe196, Glu200, Phe280, Lys197
**2** (Catalytic inhibition mode)	–6.7	3	Met258, Gly183, Trp179	Arg24, Tyr46, Ile219, Asp48, Thr263, Gln262, Val149, Gln266, Asp181
**3** (Allosteric inhibition mode)	–7.81	4	Glu200, Asn193, Ser151, Tyr152	Lys197, Phe196, Ile281, Phe280, Leu192, Ala189, Tyr153, Lys150
**3** (Catalytic inhibition mode)	–7.62	10	Asp181, Lys120, Lys116, Trp179, Gly183, Arg254, Tyr20, Arg24	Glu115, Arg221, Ser216, Tyr46, Gln262, Asp48, Gln266, Gly259, Ile219
Compound **2** (Allosteric inhibitor)	–10.98	2	Asn193, Glu276	Phe196, Gly277, Phe280, Ile281, Met282, Lys279, Ala189, Leu192
Compound **23** (Catalytic inhibitor)	–11.23	11	Asp48, Tyr46, Arg24, Ser216, Ala217, Arg221, Arg254, Gln262	Yls116, Phe182, Gln266, Gln262, Ala217, Et258, Gly259, Asp29, Ser28, Val49, Ile219, Tyr46

^a^ Estimated the biding free energy of the ligand receptor complex; ^b, c, d^ The number of hydrogen bonds and all amino acid residues from the enzyme inhibitor complex were determined with the AutoDock 4.2.6 program (The Scripps Research Institute, Molecular Graphics Laboratory, San Diego, CA, USA).

**Table 3:** Molecular interaction of the α-glucosidase active site with **1**–**3** as well as reported inhibitors.

Compound	Binding Energy ^a^ (kcal/mol)	No. of H-Bond ^b^	H-Bond Interacting Residues ^c^	Hydrophobic Interacting Residues ^d^
**1** (Allosteric inhibition mode)	−8.65	4	Ile262, Tle272, Glu296, Leu297	Arg263, Val266, Gly269, Arg270, Glu271, Thr290, Ser291, Ala292, His295
**1** (Catalytic inhibition mode)	−10.43	4	Ser240, Asp242, Leu313, Arg315	His280, Ser311, Lys156, Asp307, Thr310, Pro312, Gln279, Glu277, Val216, Phe303, Asp352, Phe314
**2** (Allosteric inhibition mode)	−11.71	3	Pro8, Arg270, Glu296	Trp15, Lys16, Thr274, Thr290, His295, Leu297, Ser298, Trp343, Cys342, Ala292, Asp341, Glu271, Gly269, Val266, Ile262, Ile272, Asn259, Arg263
**2** (Catalytic inhibition mode)	−9.48	2	Ser241, Thr306	Lys156, Ser157, Tyr158, Glu227, Ser240, Asp242, His280, Phe303, Pro312, Phe314, Arg315, Tyr316, Tyr347 Asn350, Asp352, Gln353, Glu411, Asn415
**3** (Allosteric inhibition mode)	−7.36	8	Ile272, Glu296, Thr274, Glu11, His295, Asn259, Ser298	Arg270, Ile262, Arg263, Gly269, Glu271, Lys13, Ala292, Lys16, Leu297, Trp15, The290
**3** (Catalytic inhibition mode)	−11.53	4	Ser241, Asp307, Asn415	Lys156, Tyr158, Phe178, Val216, Gln239, Ser240, Asp242, Glu277, Gln279, His280, Phe303, Phe314, Arg315, Tyr316, Asp351, Gln353, Glu411, Arg442,
BIP (Allosteric inhibitor)	−6.85	2	Glu296, His295	Asp341, Cys342, Ala292, Thr290, Arg294, Leu297, Asn259, Ser291, Ser298, Trp15, Lys16, Trp343
Acarbose (Catalytic inhibitor)	−8.60	17	Tyr158, His112, Gln182, Asp69, Asp215, Arg213, Glu277, Asp352, Arg442, Asp307, His280, Asp242, Ser240	Lys156, Gln279, Arg315, Phe178, Phe303, Gln353, Tyr72, Val216, His351, Glu411

^a^ Estimated the biding free energy of the ligand receptor complex; ^b, c, d^ The number of hydrogen bonds and all amino acid residues from the enzyme inhibitor complex were determined with the AutoDock 4.2.6 program.

## Supplementary Materials

Supplementary materials can be found at http://www.mdpi.com/1422-0067/19/5/1542/s1.

## Abbreviations

ADT	AutoDockTools
IR	Insulin resistance
PTP1B	Protein tyrosine phosphatase 1B
TCM	Traditional Chinese Medicine
T2DM	Type II diabetes mellitus
