# Supplementary material for: Protein Tyrosine Phosphatase 1B Inhibition and Glucose Uptake Potentials of Mulberrofuran G, Albanol B, and Kuwanon G from Root Bark of Morus alba L. in Insulin-Resistant HepG2 Cells: An In Vitro and In Silico Study
Source: Int J Mol Sci. 2018 May 22;19(5):1542. doi: 10.3390/ijms19051542 (PMC5983811; doi:10.3390/ijms19051542)
Supplement: Supplementary file 1 [file ijms-19-01542-s001.pdf]

International Journal of *Molecular Sciences*

MDPI

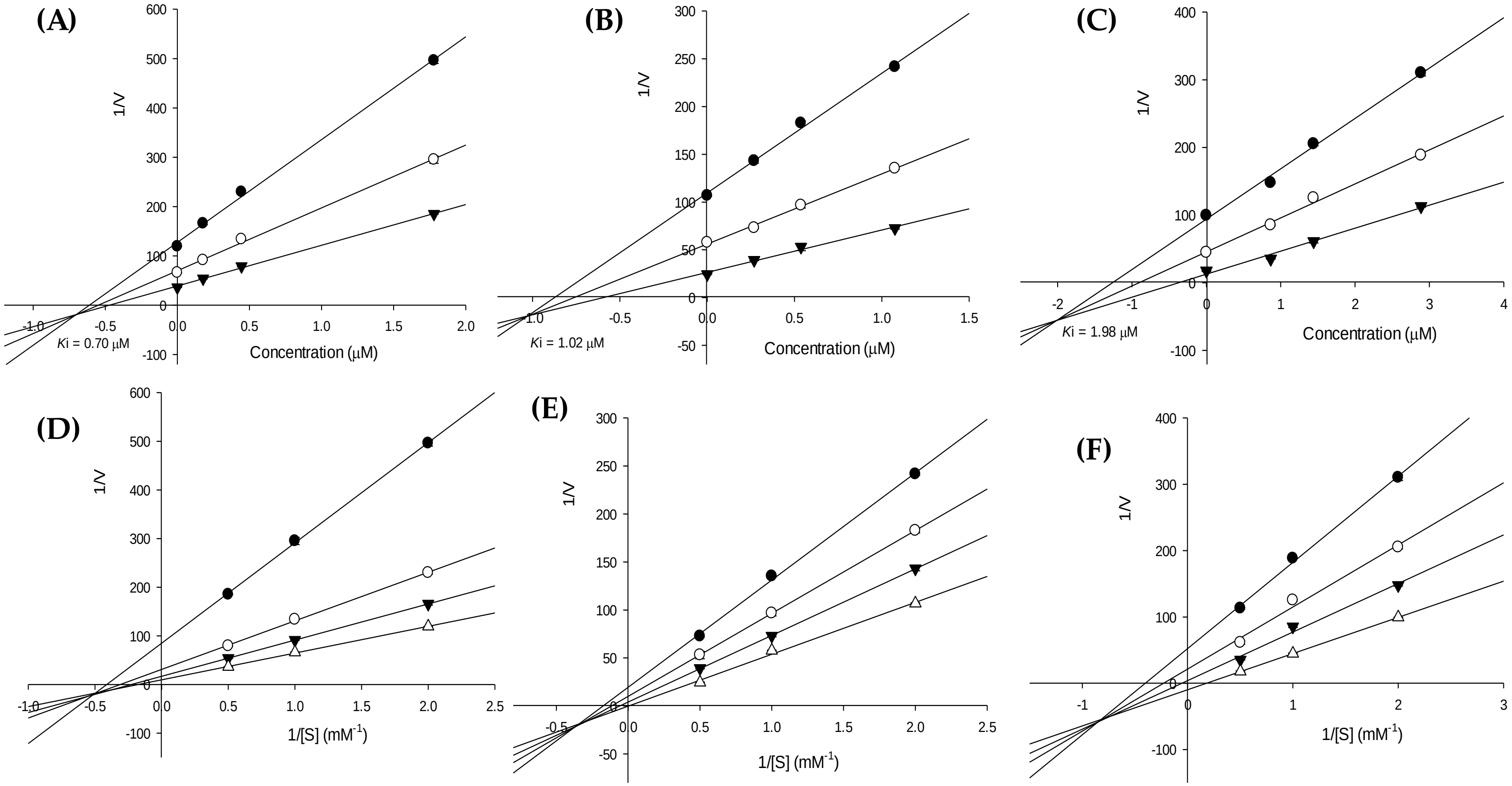

Figure S1. Enzyme kinetic plots of PTP1B inhibition by **1**-**3**. (A-C) Dixon plots of PTP1B inhibition. **1** (A), **2** (B) and **3** (C) were tested in the presence of different substrate concentrations: 0.5 mM (●), 1.0 mM (○) and 2.0 mM (▼). (D-F) Linweaver–Burk plots of PTP1B inhibition by **1**-**3**. PTP1B inhibition was analyzed in the presence of different concentrations of test samples as follows: 1.78 μM (●), 0.44 μM (○), 0.18 μM (▼), 0 μM (△) for **1**; 1.07 μM (●), 0.54 μM (○), 0.27 μM (▼), 0 μM (△) for **2**; 2.89 μM (●), 1.44 μM (○), 0.87 μM (▼), 0 μM (△) for **3**.

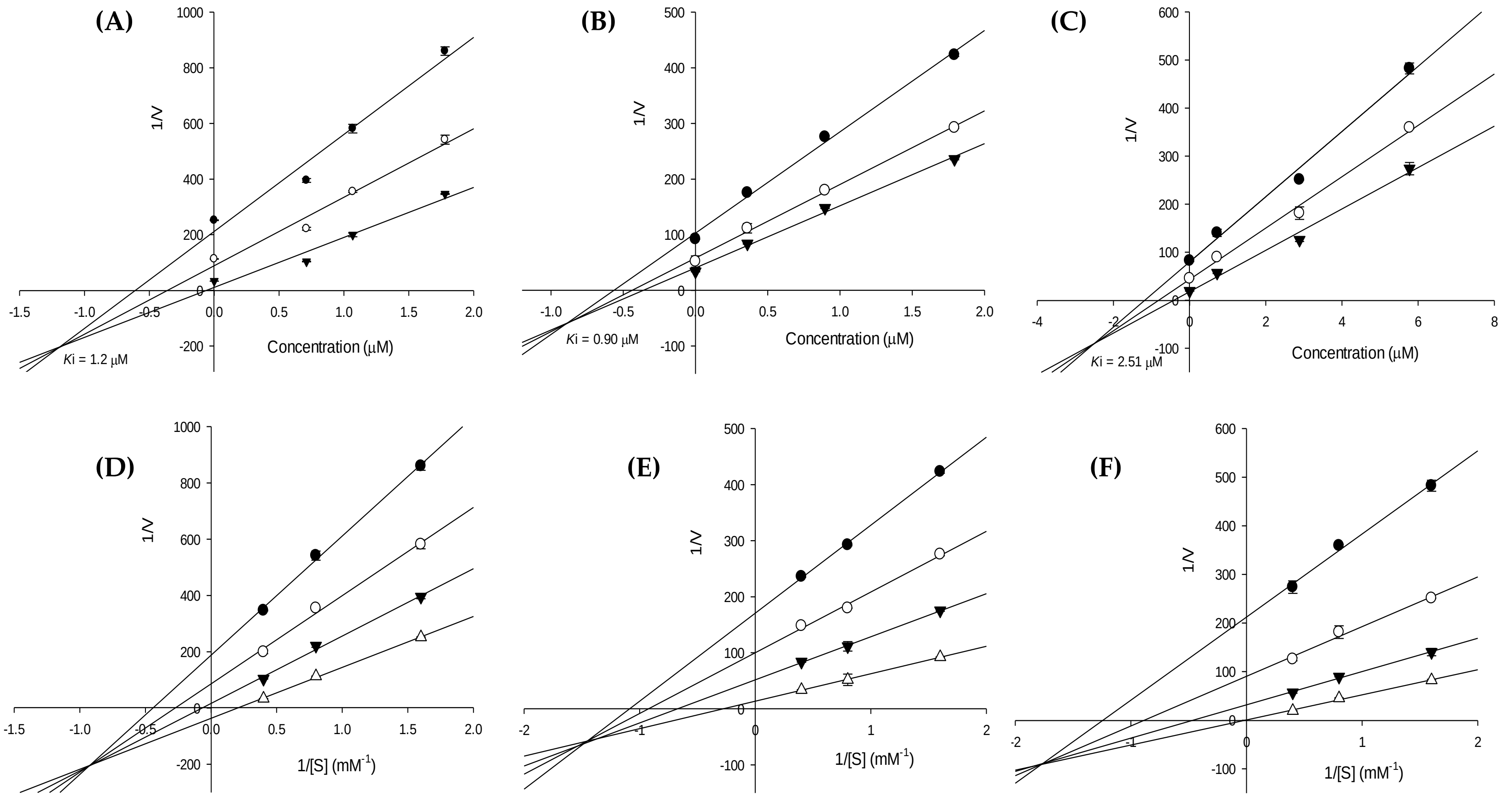

Figure S2. Enzyme kinetic plots of α-glucosidase inhibition by **1**-**3**. (A-C) Dixon plots of α-glucosidase inhibition. **1** (A), **2** (B) and **3** (C) were tested in the presence of different substrate concentrations: 0.4 mM (●), 0.8 mM (○) and 1.6 mM (▼). (D-F) Linweaver–Burk plots of α-glucosidase inhibition by **1**-**3**. α-Glucosidase inhibition was analyzed in the presence of different concentrations of test samples as follows: 1.78 μM (●), 1.07 μM (○), 0.71 μM (▼), 0 μM (△) for **1**; 1.79 μM (●), 0.9 μM (○), 0.36 μM (▼), 0 μM (△) for **2**; 5.77 μM (●), 2.88 μM (○), 0.72 μM (▼), 0 μM (△) for **3**.
